# Post-traumatic Acinetobacter baumannii Meningitis Following Penetrating Trauma: A Case Report

**DOI:** 10.7759/cureus.76235

**Published:** 2024-12-23

**Authors:** Wilhelm Hansen, Ingrid Dreyer, Lourens Botes, Karishma Domah, Rose Kashimbode

**Affiliations:** 1 Department of General Surgery, University of the Witwatersrand, Johannesburg, ZAF; 2 Department of Critical Care Medicine, Chris Hani Baragwanath Academic Hospital, Johannesburg, ZAF

**Keywords:** acinetobacter baumannii meningitis, drug-resistant, infectious diseases, neurosurgery, penetrating trauma

## Abstract

This report details the case of a 29-year-old male patient who presented at a tertiary-level trauma centre with multiple stab wounds to the face, chest, and back. Despite not undergoing surgical intervention or exhibiting any apparent cerebrospinal fluid (CSF) leakage during the initial evaluation. The patient's condition deteriorated, with subsequent cultures from CSF and blood confirmed extensively drug-resistant (XDR) Acinetobacter baumannii (A. baumannii) meningitis. Imaging revealed a spinal canal breach with abscess formation, necessitating targeted antibiotic therapy and neurosurgical debridement.

This case highlights the potential challenges in diagnosing and managing A. baumannii meningitis, especially in trauma patients. Early recognition of risk factors, thorough clinical examination and multidisciplinary care were crucial for clinical improvement. The case also highlights the critical need for robust infection control measures to address the rising threat of XDR pathogens in healthcare settings.

## Introduction

This case report addresses an instance of Acinetobacter baumannii (A. baumannii) meningitis in the context of penetrating trauma. A. baumannii has emerged as a leading cause of healthcare-associated infections, namely, of the respiratory, urinary and integumentary systems [1]. Central nervous system involvement is an increasingly more important entity with high mortality rates, often found in the setting of post-neurosurgical intervention [1,2]. Evolving antibiotic resistance and ability to persist in the environment for prolonged periods underpin the need for strict infection prevention and control measures in controlling the spread of A. baumannii [3]. This work contributes to the pool of knowledge and implications for the practice of managing multi-drug-resistant (MDR) pathogens in resource-constrained trauma settings.

## Case presentation

A 29-year-old male patient was brought by ambulance to a tertiary-level trauma centre following an alleged assault. The primary trauma survey documented 14 penetrating stab wounds involving the patient's posterior scalp and neck, as well as upper and lower back, with two paraspinal injuries at the level of C5/C6 and T11/T12. On the primary survey, the treating clinician noted no other issues and assessed the patient to have a Glasgow Coma Scale (GCS) of 15/15 with no focal neurological deficit initially noted. The initial treatment consisted of suturing the wounds, stat dose of intravenous (IV) amoxicillin/clavulanic acid and intramuscular tetanus toxoid. 

Due to the extensive trauma, computed tomography (CT) scans of the brain, cervical spine, and abdomen were done, which revealed pneumocephalus in the posterior cerebral fossa with no associated calvarial fractures or intracranial haemorrhage, a right lower lobe lung laceration with an occult haemopneumothorax, and surgical emphysema underlying the two paraspinal injuries. No bony cervical spine or intra-abdominal injuries were noted. The patient was admitted to the general trauma ward with an intercostal drain for observation and prophylactic ceftriaxone for pneumocephalus. 

**Figure 1:**
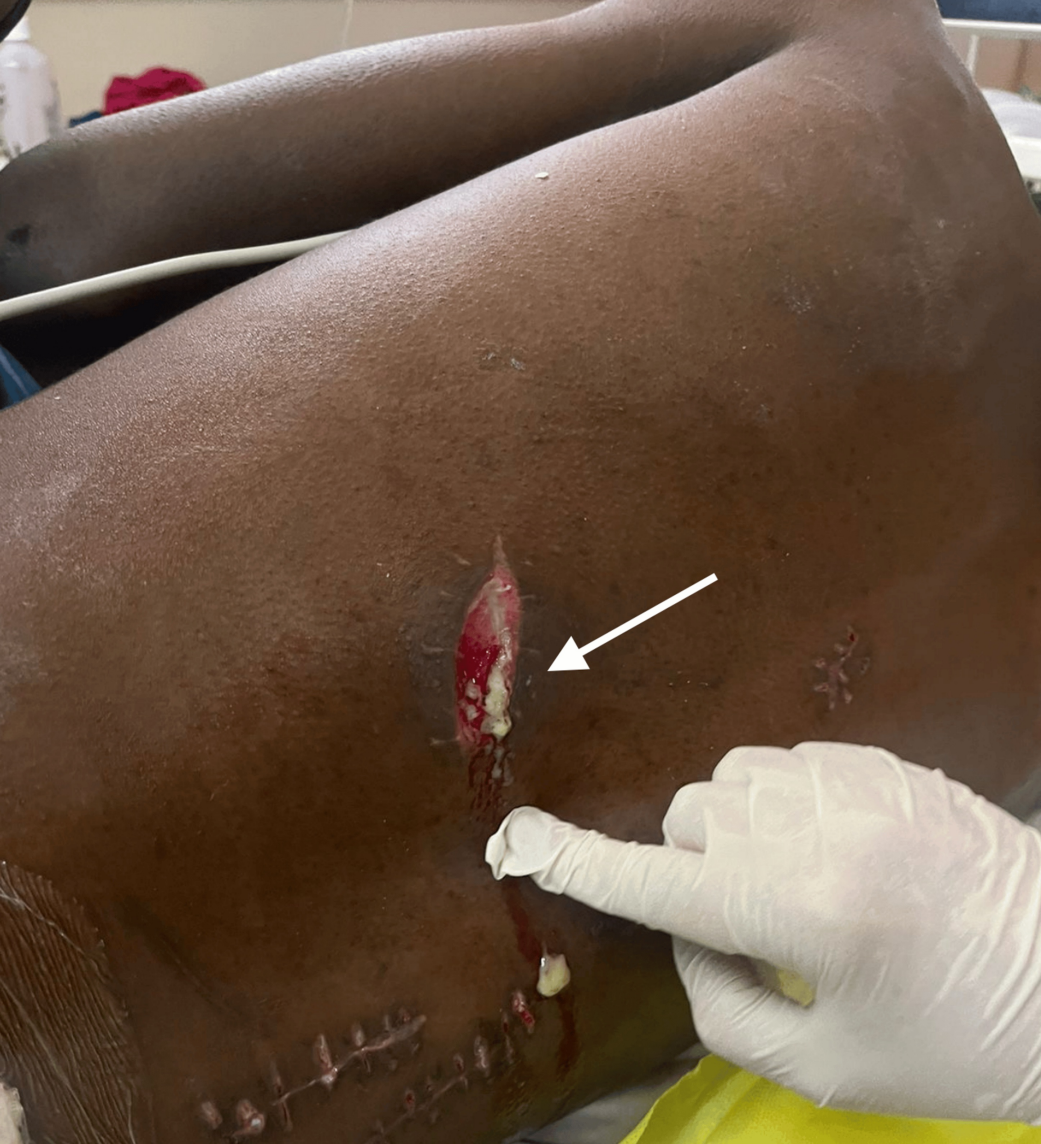
Left paraspinal stab draining pus

During the first five days in the ward, the patient was reviewed by both the trauma and neurosurgical teams, with no new neurological fallout documented. A CT angiogram of the neck was also done during this time, which excluded vascular injury. Whilst being prepared for discharge, the patient reported new-onset lower back pain and a purulent discharge was noted at the site of the lower paraspinal wound (Figure 1). 

The patient's clinical condition drastically deteriorated over the subsequent three days, ultimately necessitating intubation and an urgent CT brain scan that demonstrated cerebral oedema and features of raised intracranial pressure. A septic work-up was done before transferring the patient to the hospital's Intensive Care Unit (ICU). As part of this work-up, radiology reviewed the images, but the CT slices were too thick to exclude any blood-brain barrier breach. 

**Figure 2:**
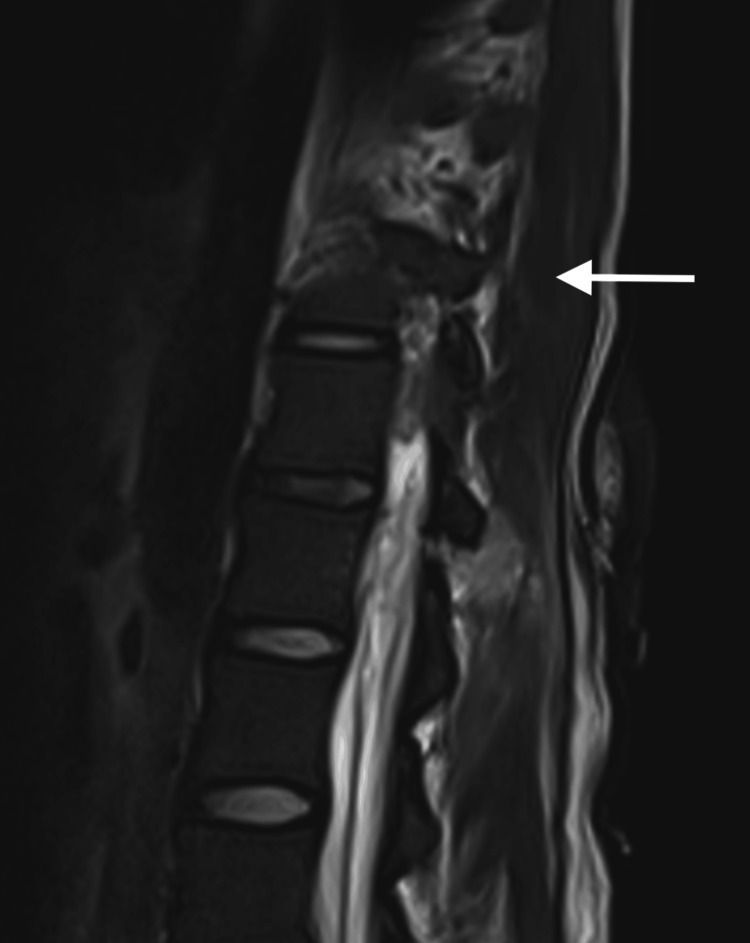
MRI T2 sagittal view of the stab at the L1 level

**Figure 3:**
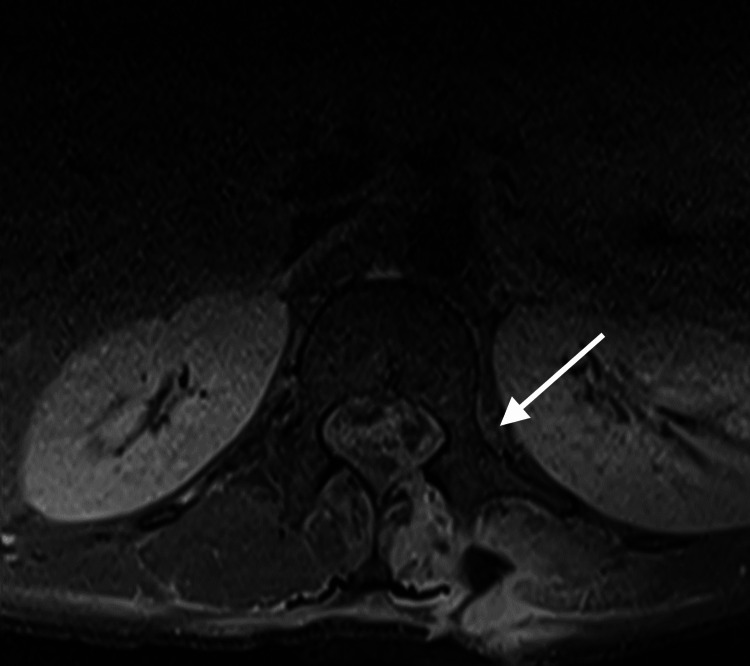
MRI T1 axial view of the stab at the L1 level

**Figure 4:**
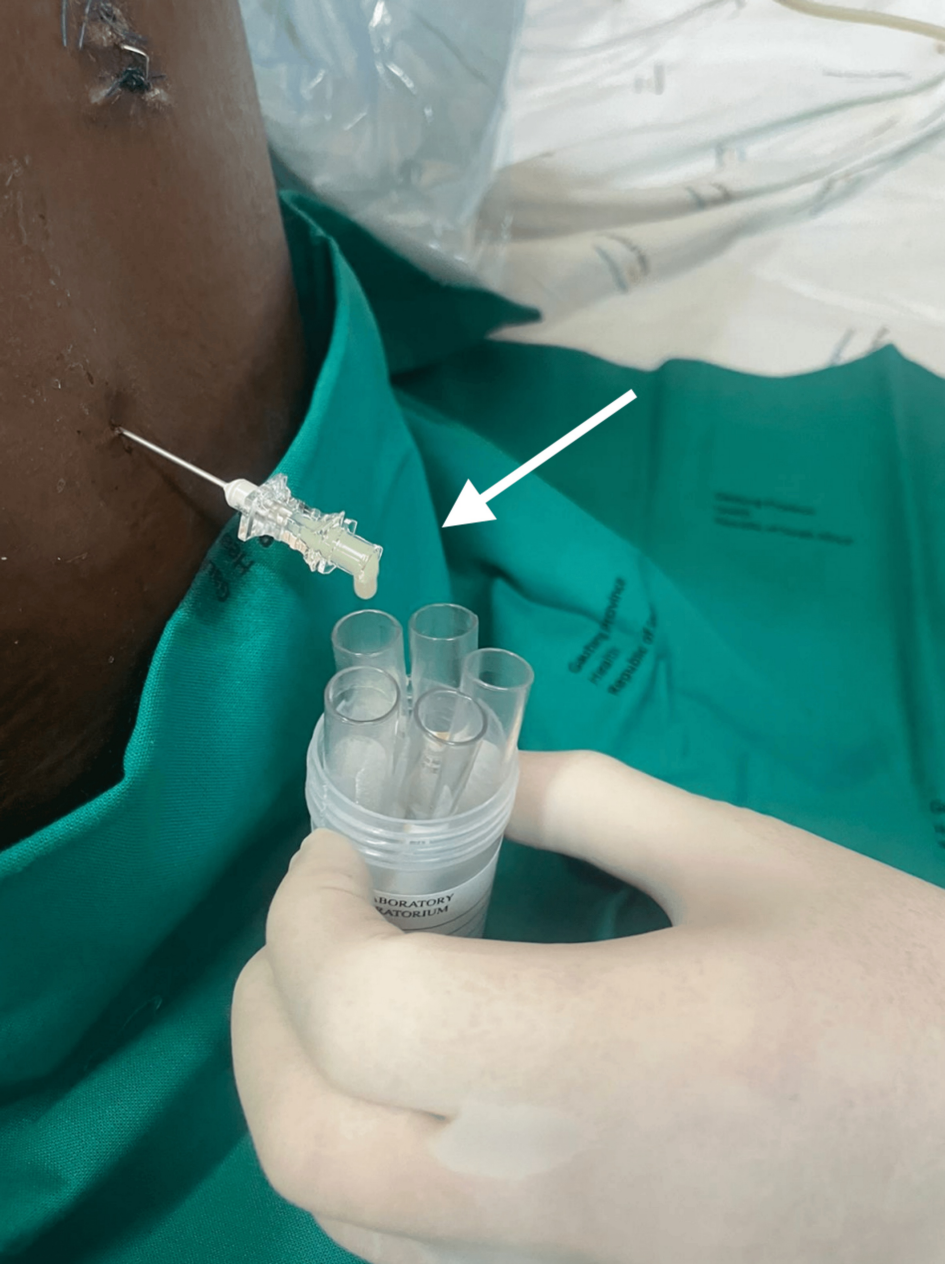
LP draining frank pus LP: Lumbar puncture

In the ICU, the patient was started on amikacin, meropenem and steroids for suspected meningitis, and a lumbar puncture (LP) was performed by the neurosurgical team. His retroviral status was also tested, and it came back negative. Furthermore, no other causes of immunocompromise were reported. Both the blood and cerebrospinal fluid (CSF) cultures flagged positive for extensively drug-resistant (XDR) A. baumannii (Table 1). Per the ICU protocol, the patient was initiated on dual-antibiotic therapy, comprising an IV polymyxin antibiotic (colistimethate sodium) and a high dose of carbapenem (meropenem). A full spinal magnetic resonance imaging (MRI) scan was done for further evaluation (Figures 2, 3). The MRI scan indicated a stab tract of 5.6 cm deep at the left paraspinal L1 vertebral body level, breaking through the left L1 lamina to communicate with the spinal canal and cause partial transection of the left conus medullaris and splaying of the proximal cauda equina. An associated lumbar spine abscess extending from L1 to L5 with intradural extension was also noted. Despite the initiation of targeted antibiotics, the patient's clinical condition did not improve, with a persistently decreased GCS of 7T/10 (E2M5VT) and a new left-sided hemiparesis noted. A repeat LP yielded frank pus (Figure 4). 

**Table 1:** Results CRP: C-reactive protein, PCT: procalcitonin, WCC: white cell count, XDR: extensive drug-resistant, MIC: minimum inhibitory concentration, GNB: gram-negative bacilli, HIV: human immunodeficiency virus

Day of Hospital Stay	Specimen Type	Result	Reference Range
Day 7	Blood	CRP: 61	<10 mg/L
		PCT: 0.06	0.0-0.05 ng/ml
		WCC: 8.04	3.92-10.40 x 10^9^/L
Day 10	Blood culture	XDR A baumannii	-
		Colistin MIC 0.5 ug/mL	-
Day 11	Blood culture	XDR A baumannii	-
		Colistin MIC 1.0 ug/mL	-
Day 13	Lumbar puncture	Gram stain: GNB observed	-
		India Ink stain: encapsulated yeasts not observed	-
		Cryptococcal antigen: negative	-
		Culture: XDR A baumanii	-
Day 14	Blood	CRP: 235	<10 mg/L
		PCT: 20.58	0.0-0.05 ng/ml
		WCC: 4.62	3.92-10.40 x 10^9^/L
Day 16	Blood	HIV negative	-
Day 16	Lumbar puncture	Gram stain: GNB observed	-
		Culture: XDR A baumanii	-
Day 35	Blood	CRP: 11	<10 mg/L
		PCT: 0.07	0.0-0.05 ng/ml
		WCC: 6.01	3.92-10.40 x 10^9^/L

Neurosurgery took the patient to theatre for wound debridement, laminectomy of T12-L1 and evacuation of the intradural abscess 19 days after the initial injury. The patient returned to the ICU post-operatively and continued to receive targeted IV antibiotics. 

The patient was ultimately extubated and discharged to the neurosurgical ward with a documented GCS of 12/15 (E4V2M6) and left hemiplegia 23 days after the initial injury. Ten days after his ICU discharge, the patient was followed up in the ward and found to be doing well, participating in daily physical and occupational therapy. Upon further review in the ward, it was noted that he had improved septic markers with improvement in his (motor) power. Before being discharged home, he had a GCS 15/15 with full power on the left and power 4/5 in the left upper limb and 4/5 in the left lower limb. The patient subjectively reported significant improvement in his power and functionality. He completed a 21-day course of antibiotics and was followed up at the neurosurgery outpatient department.

## Discussion

Acinetobacter spp are gram-negative coccobacilli that lack motility, require oxygen for growth, test positive for catalase activity and are oxidase negative [4,5]. Of these, A. baumannii has emerged from a relatively unknown pathogenicity organism to an infectious agent that often exhibits an MDR or XDR pattern in hospitals worldwide. An essential characteristic of the organism is its ability to tolerate desiccation, which leads to its presence in diverse locations in the hospital, including ICUs. Its transmission can range from skin carriage to contamination of the environment and medical equipment [6]. Through this, it can colonise the respiratory tract, skin, urinary tract, and wounds. However, ventilator-associated pneumonia and bloodstream infections are the most commonly observed clinical manifestations of Acinetobacter infections [4,6].

According to Korinek et al., risk factors for Acinetobacter meningitis are CSF leakage, concomitant incision infection, prolonged duration of surgery, surgery that enters a sinus, increased severity of illness, prolonged external ventricular drainage, and the need for repeat surgery [7]. However, most of these risk factors cannot be applied to the patient in the case presented. Identifiable risk factors in this patient are CSF leakage secondary to penetrating injuries and concomitant incision infection.

Pyrexia and a change in the level of consciousness are typical findings of A. baumannii meningitis, whereas meningeal signs, focal neurological signs, and seizures are relatively uncommon. In these cases, the CSF presents with an increased white blood cell count (of which 75-100% are polymorphonuclear cells), raised protein, and either a normal or decreased glucose level [8,9].

Colistin and other polymyxins are often used to treat MDR A. baumannii. However, their penetration into the CSF is not well understood, partly due to challenges in measuring the concentrations of these antibiotics. It is theorised that it does not consistently cross the blood-brain barrier in non-inflamed meninges. Colistin is administered intravenously as colistin methanesulfonate (CMS), an inactive pro-drug, complicating the measurement of active colistin in the CSF. Limited studies indicate that the maximum CSF concentration of 1.25 µg/mL occurs one hour after administering 1 million International Units (80 mg) of CMS [10].

Reports on IV CMS in treating Acinetobacter meningitis are mixed. In some cases, IV CMS alone cured patients, with dosages varying from 225 mg every eight hours to 1.25 mg/kg every six hours [2,10]. However, there are instances where IV CMS failed, and CSF sterilisation was only achieved with intraventricular administration of the drug.

Given the uncertainty about the penetration of CMS and colistin into the CSF, relying solely on IV CMS for treating Acinetobacter meningitis may not be advisable. Intraventricular or intrathecal administration of polymyxins has shown promising results. The Infectious Diseases Society of America (IDSA) guidelines recommend antibiotic therapy for 21 days in gram-negative meningitis [11].

Karaiskos et al. reported that intraventricular and intrathecal colistin was a safe and successful mode of therapy for treating MDR A. baumannii meningitis [2]. However, this option was not feasible in this case after a discussion with the neurosurgical department, as the intradural abscess could become loculated and thus lead to inadequate penetration of antibiotics. Lu et al. identified in their gram-negative bacillary meningitis study in adult post-neurosurgical patients that early initiation of appropriate antibiotics led to a better outcome [12]. The literature describes a mortality rate for A. baumannii meningitis ranging from 15 to 71% [2].

## Conclusions

This case report highlights the uncommon occurrence and the challenges involved in managing A. baumannii meningitis, which is becoming an increasingly common clinical entity, particularly in neurosurgery and craniomaxillofacial trauma cases. These infections are often MDR, and colonisation of respiratory secretions, urine, or wounds frequently precedes nosocomial cases. Strict infection control measures are paramount to prevent the dissemination of the infection. Moreover, should antibiotics exhibit inadequate absorption into the CSF through IV administration, it is advisable to consider intraventricular administration to attain bactericidal concentrations and ensure the complete eradication of the infection.

It is essential to thoroughly examine the patient from head to toe, with complete exposure. Clinicians must recognise that paraspinal stab wounds possess the potential to compromise the spinal cord and its protective meninges. In light of the trajectory associated with these injuries, it is essential to conduct a thorough assessment of potential specific injuries. Additionally, if a patient's condition changes and clinical findings are incongruent, obtaining appropriate imaging is imperative to aid in accurate diagnosis.
